# A rare spontaneous breast abscess due to *Mycobacterium chelonae*: a case report

**DOI:** 10.1186/s40792-023-01706-8

**Published:** 2023-07-05

**Authors:** Yayoi Sakatoku, Yoshito Okada, Yohei Takahashi

**Affiliations:** 1 Department of Surgery, Handa City Hospital, 2-29 Toyo-Cho, Handa-City, Aichi 475-8599 Japan; 2 Department of Diagnostic Pathology, Handa City Hospital, 2-29 Toyo-Cho, Handa-City, Aichi 475-8599 Japan

**Keywords:** *Mycobacterium chelonae*, Breast abscess, Nontuberculous mycobacteria, Case report

## Abstract

**Background:**

*Mycobacterium chelonae*, a nontuberculous mycobacterium, commonly causes skin, soft tissue, eye, pulmonary, catheter-related, and post-surgical infections in patients with immunosuppression or trauma. *M. chelonae* breast infections are rare, and most cases occur following cosmetic surgery. Here, we report the first case of spontaneous breast abscess due to *M. chelonae*.

**Case presentation:**

A 22-year-old Japanese woman presented at our hospital with swelling and pain in the right breast for the past 2 weeks without any fever. She had a 19-month-old child and stopped breastfeeding 1 month after giving birth. The patient had no history of trauma or breast surgeries, no family history of breast cancer, and was not immunocompromised. Breast ultrasonography revealed a heterogeneous hypoechoic lesion with multiple fluid-filled areas suspected to be abscesses. Dynamic contrast-enhanced magnetic resonance imaging revealed a 64 × 58 × 62 mm, ill-defined, high-signal-intensity lesion with multiple ring enhancements in the upper half of the right breast. The first diagnosis was inflammatory breast cancer or granulomatous mastitis with abscess. A core needle biopsy led to drainage of pus. Gram staining did not reveal any bacteria in the pus, but the colonies from the biopsy grew on blood and chocolate agar cultures. Mass spectrometry detected *M. chelonae* in these colonies. Histopathological findings revealed mastitis without malignancy. The patient's treatment regimen was oral clarithromycin (CAM) based on susceptibility. Three weeks later, although the pus had reduced, the induration in the breast did not resolve; therefore, multidrug antibiotic treatment was initiated. The patient received amikacin and imipenem infusion therapy for 2 weeks, followed by continuation of CAM. Three weeks later, tenderness in the right breast recurred with slight pus discharge. Hence, minocycline (MINO) was added to the treatment. The patient stopped CAM and MINO treatment 2 weeks later. There was no recurrence 2 years after treatment.

**Conclusion:**

We report a case of *M. chelonae* breast infection and abscess formation in a 22-year-old Japanese woman without obvious risk factors. *M. chelonae* infection should be considered in cases of intractable breast abscess, even in patients without immunosuppression or trauma.

## Background

*Mycobacterium chelonae* is a nontuberculous mycobacterium (NTM), ubiquitous in the environment and found in soil, water, and aquatic animals. It is reported to cause infection of the lungs, skin/soft tissues, cornea, and bone marrow, especially in patients with trauma or immunodeficiencies [1]. Few reports exist on breast abscesses caused by *M. chelonae*, and most are associated with breast augmentation or reduction mammoplasty [2–5]. To the best of our knowledge, there is no report of spontaneous breast abscess due to *M. chelonae*.

Here, we report a case of a 22-year-old Japanese woman with no obvious risk factors, who developed a breast abscess due to *M. chelonae* infection.

## Case presentation

A 22-year-old Japanese woman presented to our hospital with swelling and pain in the right breast for the past 2 weeks without any fever. She had a 19-month-old child and had not breastfed recently. The patient had no history of trauma or surgeries related to the breast, or no family history of breast cancer and was not immunocompromised.

**Fig. 1:**
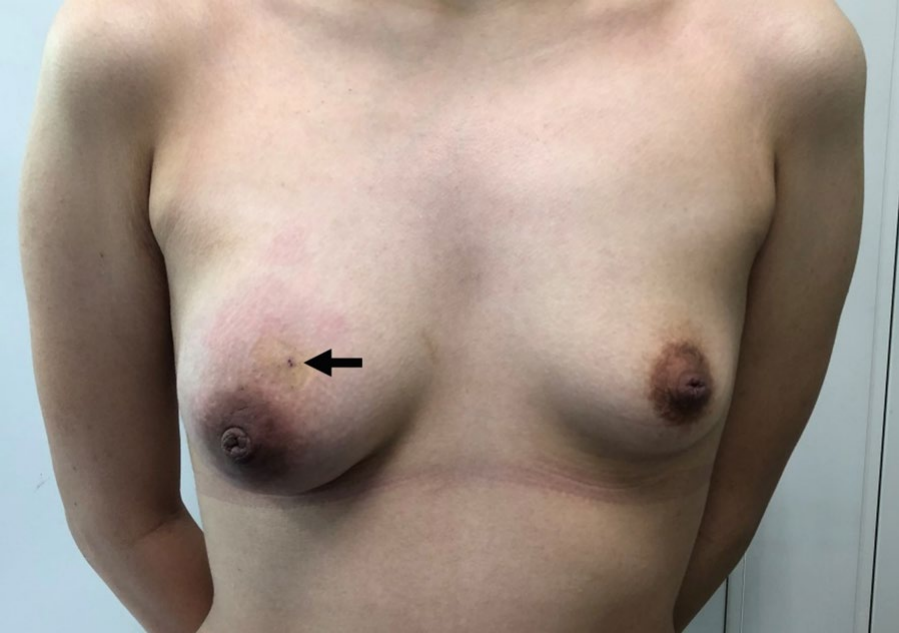
Physical examination of patient’s breasts. Marked swelling accompanied by mild redness on the right breast; core needle biopsy scar (arrow)

**Fig. 2:**
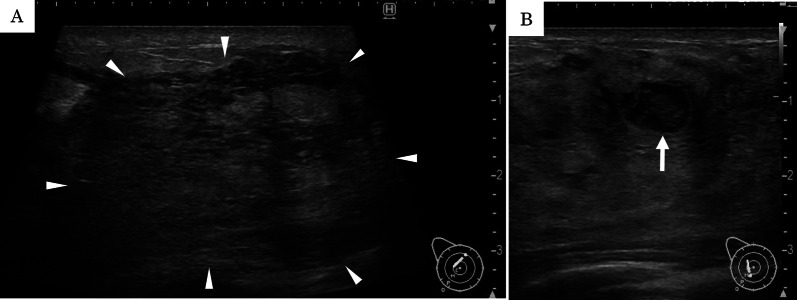
B-mode gray ultrasonography of the right breast. **A** Partially heterogeneous hypoechoic lesion (arrowhead). **B** Presence of a cyst suspected abscess (arrow)

**Fig. 3:**
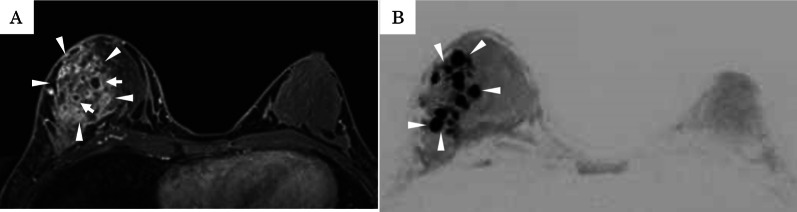
Dynamic contrast-enhanced magnetic resonance imaging: coronal view of the right breast. **A** T1-weighted image shows a 64 × 58 × 62 mm ill-defined high signal intensity lesion (arrowhead) with multiple ring enhancements in the upper half of the right breast (arrow). **B** Diffusion-weighted imaging shows strong diffusion restriction (arrowhead)

**Fig. 4:**
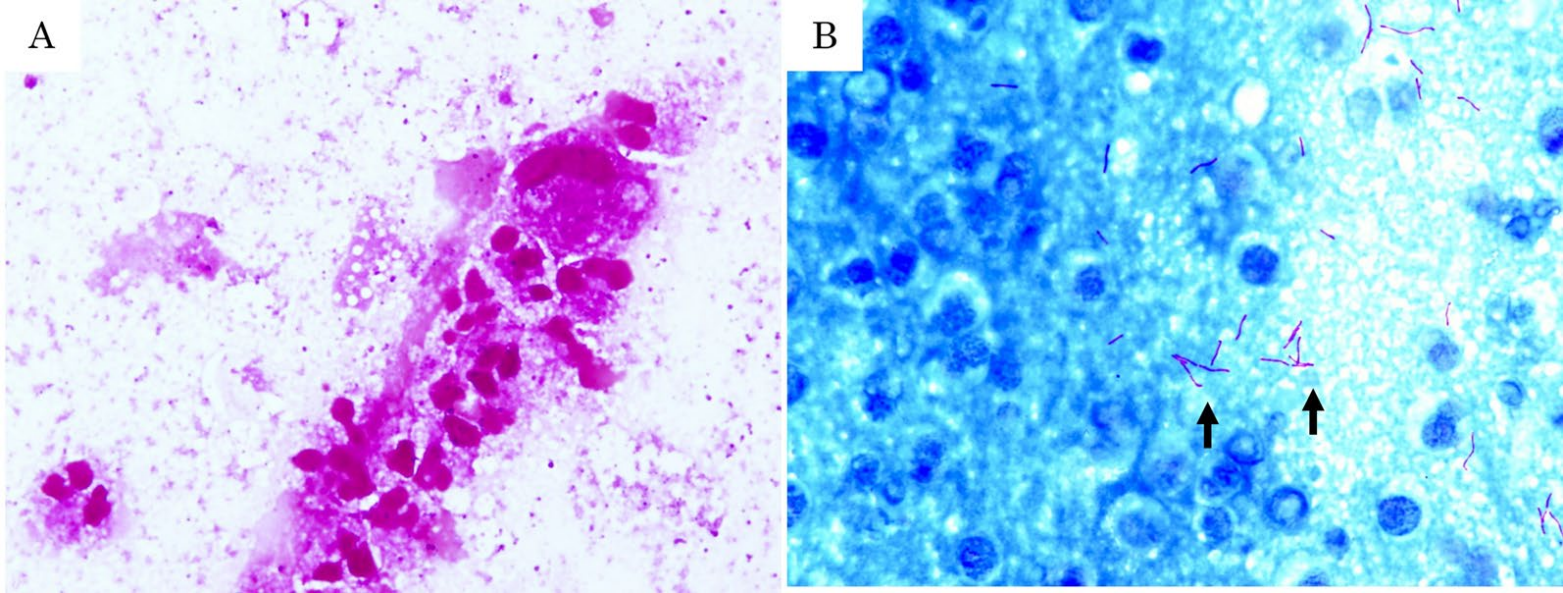
Microscopic findings of the pus samples. **A** Gram staining showing only white blood cells. Gram-positive bacilli were not detected (× 20). **B** Ziehl–Neelsen staining showing many rod-shaped acid-fast bacilli (arrow) (× 20)

**Fig. 5:**
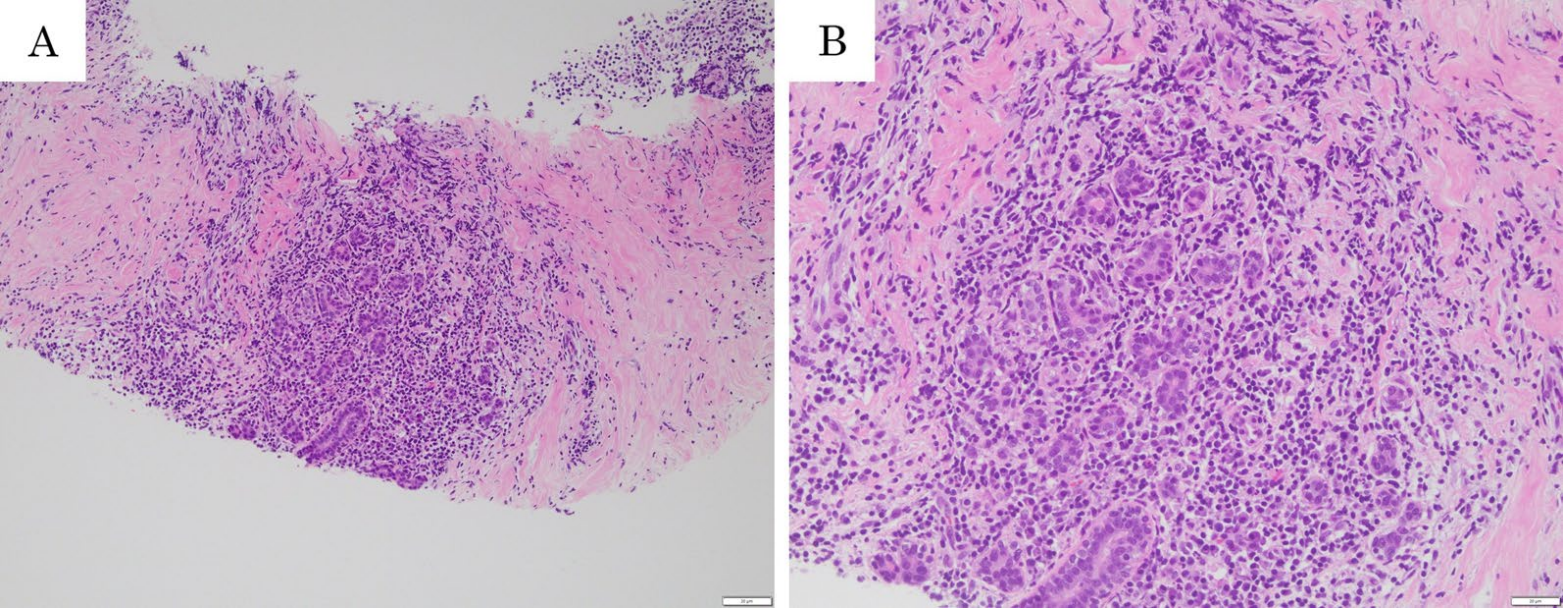
Histopathological findings. Histopathological findings by core needle biopsy show infiltration of inflammatory cells (neutrophils and lymphocytes, and mammary gland tissue with infiltration of foam cells) **A** Hematoxylin and eosin [H.E.] staining, lower magnified image × 100; **B** H.E. staining, higher magnified image, × 200). No granulomatous changes or malignant findings are observed

Physical examination revealed a palpable tender mass with swelling in the upper half of the right breast (Fig. 1). A routine breast ultrasonography revealed a heterogeneous hypoechoic lesion with multiple fluid-filled areas, suspected to be abscesses (Fig. 2A, B). Dynamic contrast-enhanced magnetic resonance imaging (DCE-MRI) revealed a 64 × 58 × 62 mm, ill-defined, high-signal-intensity lesion with multiple ring enhancements in the upper half of the right breast (Fig. 3A). The enhancement area showed a fast plateau pattern. Diffusion-weighted imaging on MRI showed strong diffusion restriction at the lesion (Fig. 3B). The first diagnosis based on clinical and imaging findings was inflammatory breast cancer or granulomatous mastitis with abscess. A core needle biopsy led to drainage of pus. Gram staining of the pus did not reveal any bacteria (Fig. 4A). However, the pus cultured on blood and chocolate agar plates showed the formation of bacterial colonies after 3 days of incubation. *M. chelonae*, and not *Corynebacterium,* was detected by mass spectrometry. Ziehl–Neelsen staining of the second biopsy drainage sample revealed acid-fast bacillus consistent with NTM (Fig. 4B). The susceptibility test indicated resistance to sulfamethoxazole-trimethoprim (ST) combination product, and sensitivity to amikacin (AMK), tobramycin (TOB), imipenem (IPM), and clarithromycin (CAM) (Table 1). Histopathological findings revealed mastitis in the mammary gland tissue with infiltration of inflammatory cells (neutrophils, lymphocytes and foam cells). Owing to the absence of malignancy and granulomatous lesion, inflammatory breast cancer and granulomatous mastitis were ruled out (Fig. 5A, B). No lesions in organs other than the mammary gland were observed, and screening tests for autoimmune disorders yielded negative results (Table 2).

**Table 1:** Minimum inhibitory concentration of various antibiotics on *Mycobacterium chelonae*

	MIC (mg/L)	Interpretation result
Amikacin	8	S
ST	> 2	R
Tobramycin	1	S
Imipenem	4	S
Clarithromycin	0.12	S

The susceptibility test of *M. chelonae* indicates resistance to sulfamethoxazole–trimethoprim, and sensitivity to amikacin, tobramycin, imipenem, and clarithromycin

**Table 2:** Blood examination findings before treatment

Results of blood examinations		
Complete blood counts	Reference range	Results
White blood cells, cells/μL	4500–8500	10,900
Neutrophils, %	40–69	72.1
Lymphocytes, %	19–47	22.2
Hemoglobin, g/dL	12–16	11.1
Platelets, 10^4^ counts/μL	12–30	25.3
*Serum*		
AST, U/L	13–33	11
ALT, U/L	6–27	7
Creatinine, mg/dL	0.40–0.70	0.62
IgG, mg/dL	870–1700	1290
IgA, mg/dL	110–410	275
IgM, mg/dL	35–220	227
IgG4, mg/dL	11–121	7.3
Erythrocyte sedimentation rate		
30 min, mm		20
60 min, mm	3–15	58
T-SPOT		Negative
ANA		Negative
PR3-ANCA, U/mL	0–3.5	< 1.0
MPO-ANCA, U/mL	0–3.5	< 1.0
Rheumatoid factor, U/mL	0–15	5
Angiotensin converting enzyme, U/L	8.3–21.4	9
Anti-SS-A/Ro antibody, U/mL		Negative
Prolactin, ng/mL		5.88
HIV-antibody		Negative

Blood examination findings show normal findings, except for slight elevation of inflammatory reaction, with no findings suggestive of collagen disease or immunodeficiency

Treatment included a drainage tube and oral administration of levofloxacin (LVFX). Since granulomatous mastitis was suspected based on the findings of DCE-MRI performed 3 days later, LVFX was changed to minocycline (MINO) in consideration of *Corynebacterium* infection. After 7 days, the abscess worsened and was drained additionally. Ten days later, *M. chelonae* was detected and the antibiotic was changed to CAM according to susceptibility. Multiple antibiotic therapy based on sensitivity was recommended; however, the patient rejected inpatient treatment due to family circumstances. Three weeks later, the pus discharge had reduced, and the drainage tube was removed. Although CAM administration continued, the induration in the breast did not resolve; therefore, multidrug antibiotic treatment was initiated. The patient preferred avoiding a long hospital stay, so she received AMK and IPM infusion therapy for 2 weeks, followed by continuation of CAM. Three weeks later, tenderness with slight pus discharge in the right breast recurred. Gram and Ziehl–Neelsen staining of the pus did not reveal any bacteria. Although multidrug antibiotic treatment was re-proposed, the patient requested oral antibiotics. Hence, MINO, a general antibiotic to treat NTM, was added to the treatment. Resection was proposed for residual induration, but she preferred a follow-up. She stopped taking both CAM and MINO 2 weeks later, after which, the induration gradually disappeared. There was no recurrence 2 years after treatment.

## Discussion

*M. chelonae* is a rapidly growing NTM that usually only causes infections in patients with trauma or immunodeficiencies [1]. Although NTM causes mastitis in bovines and goats, it rarely does so in humans [6–10]. There are very few reports of breast abscesses caused by *M. chelonae* following breast augmentation or reduction mammoplasty [2–5]. We were not able to find any reports of spontaneous breast abscess caused by *M. chelonae*. To our knowledge, this is the first case report of a spontaneous breast abscess due to *M. chelonae*.

NTM infections are not usually diagnosed without a strong suspicion; NTM infections cannot be detected by standard tests like Gram staining. Ziehl–Neelsen staining is a key method for diagnosing NTM. In fact, some reported cases of breast abscess caused by *M. abscessus* took several years to be diagnosed due to lack of suspicion [8, 11]. In cases where Gram staining fails to detect bacteria, repeated mastitis occurs, and there is a lack of response to conventional antibiotic treatment, NTM infection should be considered [6, 7]. Treatment for breast implant infection from NTM is removal of the implant [1]. *M. chelonae* is usually resistant to cefoxitin, and the antibiotics effective against it are IPM, TOB, AMK, CAM, linezolid, clofazimine, doxycycline, ciprofloxacin [1]. In recent years, CAM-resistant *M. chelonae* has been reported, making susceptibility testing an essential step for effective antibiotic treatment [4]. Although lung and widespread infections are treated with a combination of two or more antibiotics for a duration of at least 6 months, CAM remains the recommended treatment for localized skin infections [1]. Drainage and debridement are required in addition to antibiotics [1].

Granulomatous mastitis is a rare, benign, inflammatory disease characterized by multiple breast abscesses. It predominantly occurs in premenopausal women shortly after their last childbirth [12, 13]. The etiology of granulomatous mastitis is unknown; however, it is associated with autoimmune disorders and hyperprolactinemia [14, 15]. Recently *Corynebacteria* are recognized as pathogens in granulomatous mastitis [16–18], although they are also present in the normal bacterial flora of the skin, mucosa, and intestine [17]. *Corynebacteria* are not commonly identified using standard tests; thus, it is necessary to culture the bacteria in lipid-supplemented mediums or long-term culture. Mass spectrometry, API CORYNE kit or polymerase chain reaction are the methods used for *Corynebacterium* identification [17]. As *Corynebacterium* is lipophilic, liposoluble antibiotics such as tetracycline, macrolide, and quinolones are effective treatment options [19].

In this case, the first diagnosis based on clinical examination and DCE-MRI was granulomatous mastitis; the pus was cultured on blood and chocolate agar for trying to detect *Corynebacterium*, which is the known pathogen of granulomatous mastitis. Colonies were formed on both mediums on the third day but *M. chelonae*, not *Corynebacterium,* was detected on mass spectrometry.

At our hospital, bacterial pus is usually cultured for 2 days using both blood and chocolate agar plates. When colonies are formed on these plates, identification and antimicrobial susceptibility testing (Sysmex, Kobe, Japan) of pathogens is routinely performed. In this case, we informed the bacteriological laboratory that *Corynebacterium* was suspected. Therefore, the patient specimen was scheduled to be cultured for a longer period than usual; however, colonies formed on the third day. Only in this case, mass spectrometry was specially performed to detect *Corynebacterium*. As a result, *M. chelonae* was detected instead of *Corynebacterium*. Since we cooperated with a bacteriological laboratory, we were fortunately able to detect *M. chelonae*.

Histopathological findings revealed mastitis without granulomatous lesion and malignancy. The route of infection was unknown, and no immunological abnormalities were observed. Thus, the patient was diagnosed with spontaneous breast abscess due to *M. chelonae*.

Multiple antibiotics therapy based on sensitivity was the first choice of therapy; however, the patient rejected inpatient treatment. Therefore, only CAM was administered orally. Although the breast abscess showed improvement after drainage and oral administration of CAM, induration persisted. IPM and AMK were then administered for 2 weeks, after which CAM administration continued. However, relapse occurred 3 weeks later with tenderness and slight pus discharge. Although multidrug antibiotic treatment was re-proposed for reducing the pus discharge, she requested oral administration. Hence, MINO, a general antibiotic to treat NTM, was added to the treatment, but induration persisted. A resection was recommended, but she requested follow-up and antibiotic treatment was discontinued. The induration gradually disappeared and did not recur during the 2 years of follow-up. The route of infection with *M. chelonae* was unknown because of no lactation or history of breast surgery, breast trauma, or immune deficiencies; a clinical diagnosis of a spontaneous breast abscess due to *M. chelonae* was made. However, there may be an unknown immunocompromising factor in the background, and careful follow-up is needed.

## Conclusions

There are two possibilities for the lack of reported cases of mastitis caused by *Mycobacteria*. First, *Mycobacteria* rarely cause mastitis in healthy women, and second, it may have been overlooked because *Mycobacteria* are difficult to detect without strong suspicion. Fortunately, we were able to detect *M. chelonae* in lieu of *Corynebacterium* as the causative pathogen of granulomatous mastitis. In future cases of breast abscesses that do not respond to standard antibiotic therapy, NTM infection should be considered. When NTM is detected, multidrug and long-duration antibiotic treatment with drainage should be considered based on susceptibility. However, there is a lack of consensus on the combination of drugs used and administration period to treat NTM infection. Future studies should address these gaps in the knowledge to develop better treatment and management guidelines.

## Data Availability

All data generated during this study are included in the published article and its additional files.

## Abbreviations

NTM: Nontuberculous bacterium
*M. **chelonae*: *Mycobacterium* *chelonae*
*M. **abscessus*: *Mycobacterium* *abscessus*
DCE-MRI: Dynamic contrast-enhanced magnetic resonance imaging
MRI: Magnetic resonance imaging
ST: Sulfamethoxazole-trimethoprim
AMK: Amikacin
TOB: Tobramycin
IPM: Imipenem
CAM: Clarithromycin
LVFX: Levofloxacin
MINO: Minocycline
*M. **fortuitum*: *Mycobacterium* *fortuitum*
*M. **gordonae*: *Mycobacterium* *gordonae*
MIC: Minimum inhibitory concentration
S: Susceptible
R: Resistant
AST: Aspartate aminotransferase
ALT: Alanine aminotransferase
ANCA: Anti-neutrophil cytoplasmic antibodies
MPO: Myeloperoxidase
PR3: Proteinase3
ACE: Angiotensin converting enzyme
HIV: Human immunodeficiency virus
